# The Role of Extracting Solvents in the Recovery of Polyphenols from Green Tea and Its Antiradical Activity Supported by Principal Component Analysis

**DOI:** 10.3390/molecules25092173

**Published:** 2020-05-06

**Authors:** Wojciech Koch, Wirginia Kukuła-Koch, Marcin Czop, Paweł Helon, Ewelina Gumbarewicz

**Affiliations:** 1 Chair and Department of Food and Nutrition, Medical University of Lublin, 4a Chodźki Str., 20-093 Lublin, Poland; 2 Chair and Department of Pharmacognosy, Medical University of Lublin, 1 Chodźki Str., 20-093 Lublin, Poland; virginia.kukula@gmail.com; 3 Department of Clinical Genetics, Medical University of Lublin, Radziwiłłowska 11 Str., 20-080 Lublin, Poland; marcin.czop@umlub.pl; 4 Branch in Sandomierz, Jan Kochanowski University in Kielce, Schinzla 13a Str., 27-600 Sandomierz, Poland; phelon@ujk.edu.pl; 5 Department of Biochemistry and Molecular Biology, Medical University of Lublin, 1 Chodźki Str., 20-093 Lublin, Poland; ewelina.gumbarewicz@umlub.pl

**Keywords:** green tea, extraction, DPPH, PCA, catechins, antioxidant activity, *Camellia sinensis*, *Theaceae*

## Abstract

Green tea contains a variety of biologically active constituents that are widely used in the pharmaceutical and food industries. Among them, simple catechins constitute a major group of compounds that is primarily responsible for the high biologic activity of green tea extracts. Therefore, the application of optimized extraction conditions may result in obtaining high value extracts. The main purpose of the study was to compare the content of polyphenols, mainly catechins, and the antioxidant activity of green tea extracts obtained by three different extraction methods: simple maceration, ultrasound extraction and accelerated solvent extraction using six various solvent systems. The quality of the extracts was evaluated by LC-ESI-Q-TOF-MS methodologies and spectrophotometric determinations. The obtained results revealed that catechins’ extraction efficiency was identical for the three techniques studied. However, larger quantitative differences among the samples were observed when using different solvents. The total content of major catechins and gallic acid was within a very wide range of 10.2–842 mg/L. Ethyl acetate was by far the least effective extractant, regardless of the extraction technique used. After all, the solvent system composed of ethanol:water (1:1 *v/v*) was proven to be the best to recover catechins and to deliver extracts with the highest antiradical activity.

## 1. Introduction

Environmental toxins have a visible impact on human health and were proven to induce the formation of free radicals, the oxidation of lipids, some inflammatory conditions, marked hepatotoxicity, embryotoxicity, the progression of neurological disorders, the cell apoptosis and carcinogenesis. Among them, several hazardous factors were identified like pesticides, smoke, mycotoxins, polychlorinated biphenyls (PCBs) or arsenic, which induce the occurrence of the above-mentioned pathophysiological effects [1].

Green tea is an unoxidized and non-fermented type of tea, which is included among the world’s most widely consumed beverages. This type of tea contains a variety of polyphenols including catechins, which have shown several beneficial biological properties and have proven to be able to protect the human organism against the environmentally hazardous factors listed above. Green tea polyphenols exert significant antioxidant, anti-inflammatory, antiallergic, antimutagenic, antibacterial, antiviral, anticancer and cardioprotective properties which have been described in numerous scientific papers [2,3,4,5,6,7].

Catechins (flavan-3-ols) in terms of their structure belong to the mostly abundant group of flavonoids, which are present in a wide variety of food products of plant origin, including tea [2]. Fresh, unprocessed tea leaves contain around 36% of polyphenols, among which 30% are simple catechins. The most abundant ones, alongside with their abbreviations, are (−)-epigallocatechin gallate (EGCG), (−)-epigallocatechin (EGC), (−)-epicatechin (EC), (−)-epicatechin gallate (ECG), (−)-gallocatechin gallate (GCG), (−)-gallocatechin (GC) and (+)-catechin (C) [8]. Our previous findings [9] revealed that the first four above mentioned catechins are the major ones that are present in green tea infusions. Gallic acid (GA) in the former study was proved as the most important phenolic acid as many flavan-3-ols were esterified with this compound. All over the world, especially in Far East countries, where green tea is by far the most popular type of tea, the product is consumed as brew prepared using hot water (80–90 °C) [10,11]. However, on an industrial scale, green tea extracts are prepared using different solvents and the herein described study could add some more information on the selection of optimized conditions to increase the efficiency of catechins’ recovery process. Another parameter, which well correlates with the quality of the extract and concentration of simple flavan-3-ols, is the antioxidant activity [4,12,13]. Therefore, in vitro antioxidant activity evaluation methods may be used to complement chromatographic analysis of the extract. The main purpose of this work was to evaluate, based on PCA (Principal Component Analysis) results, the influence of extracting solvents on the content of polyphenols, mainly catechins, and the antiradical activity of green tea extracts obtained using three different extraction methods: simple maceration, ultrasound extraction and accelerated solvent extraction (ASE). The chromatographic analysis was performed using LC-ESI-Q-TOF-MS methodology to precisely identify the metabolites and determine the content of major catechins in the obtained extracts. The antiradical activity assessment was performed with the DPPH assay and the total phenolic content was determined using the Folin–Ciocalteu test. Table 1 lists the applied techniques and solvent systems used in the study together with given codes for an easier presentation of the results.

## 2. Results and Discussion

### 2.1. Qualitative Composition of the Obtained Extracts

The HPLC–MS analysis operated under the conditions described below provided clear spectra with well separated catechins from tea extracts. The further quantified catechins were eluted in the following order: GA at 3.3 min, EGC at 10.4 min, C at 11.2 min, EC at 12.5 min, EGCG at 12.9 min and ECG at 14.7 min (Table S2 Supplementary Material File). All compounds were identified with the error of measurement lower than 10 ppm.

All spectra showed a prominent peak of well-ionized citric acid at 2.5 min that was added to the extracts to adjust the pH and increase the catechins’ stability (see Table S2 in the Supplementary Material). Thanks to the purchase of two isomers: catechin and epicatechin, these compounds were undoubtedly distinguished one from the other in the obtained extracts, together with other, more complex catechins that come either from C or EC. Catechin that was better fragmented at 10 eV collision energy applied in its MS/MS spectrum showed more prominent fragments at *m/z* of 179, 165 and 125 from the compared EC. These three *m/z* signals were also visible in the MS/MS spectra of GC in our study, whereas the 165 signal was not present among ECG and EGCG fragmentation spectra. Negative ionization mode was found preferable for the quantitative analysis, also because gallic acid was visible under these conditions at much higher intensity.

### 2.2. Quantitative Composition of the Obtained Extracts

**Figure 1:**
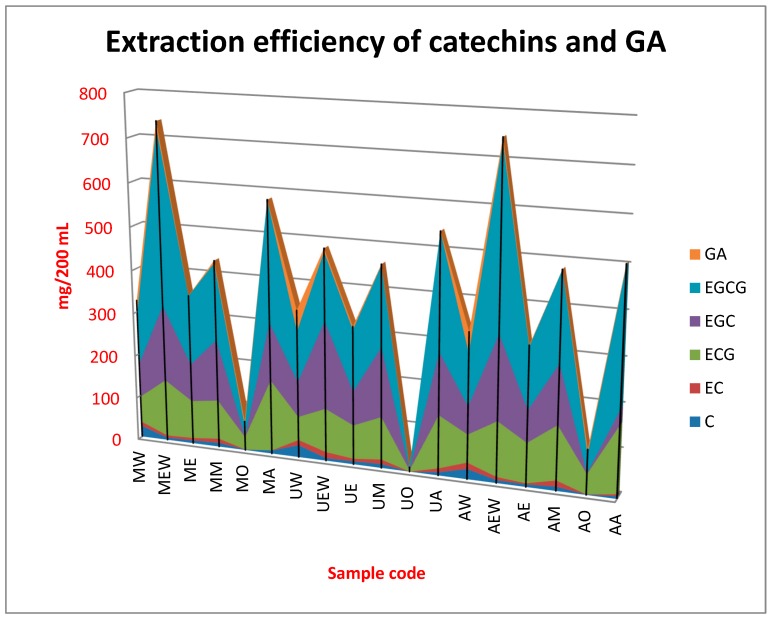
Extraction efficiency of catechins and gallic acid (GA).

Graphical illustration of the efficiency of extraction methods and each solvent system applied in the present study towards particular catechin and gallic acid was presented in Figure 1. While Table 2 presents concentration of each compound in the obtained extracts and the total amount of all investigated substances (sum of all catechins and gallic acid).

GA–gallic acid; EGCG–epigallocatechin gallate; EGC–epigallocatechin; ECG–epicatechin gallate; EC–epicatechin; C–catechin; MW–maceration with water; MEW–maceration with ethanol:water (1:1 *v/v*); ME–maceration with ethanol; MM–maceration with methanol; MO–maceration with ethyl acetate; MA–maceration with acetone:water (5:1 *v/v*); UW–ultrasound extraction with water; UEW–ultrasound extraction with ethanol:water (1:1 *v/v*); UE–ultrasound extraction with ethanol; UM–ultrasound extraction with methanol; UO–ultrasound extraction with ethyl acetate; UA–ultrasound extraction with acetone:water (5:1 *v/v*); AW–Accelerated Solvent Extraction (ASE) with water; AEW–ASE with ethanol:water (1:1 *v/v*); AE–ASE with ethanol; AM–ASE with methanol; AO–ASE with ethyl acetate; AA–ASE with acetone:water (5:1 *v/v*)

Chromatographic analysis of green tea extracts obtained by various methods and using different solvent systems revealed the presence of six main polyphenols: C, EC, ECG, EGC, EGCG and GA. EGCG was the dominant catechin, whose content significantly exceeded the concentration of other compounds. Moreover, EGC and ECG were determined in high amounts. This is in agreement with other studies suggesting these two catechins being major catechins in green and black tea [8,15,16,17]. The EGCG concentration in the obtained extracts was in a fairly wide range of 5.44–406 mg/200 mL. The lowest efficiency was obtained using ultrasounds and ethyl acetate, while the highest applying ASE and a mixture of ethanol and water. EGCG was proved to be a major catechin in all extracts, except UEW in which the dominant catechin was EGC and MO and AO in which ECG was determined in the highest concentration. The third major catechin in all extract was ECG (with the exceptions mentioned above), while the concentration of C, EC and GA was much lower in comparison to the three major compounds. Qualitative composition of the extracts was similar to catechin profile of the green tea infusions obtained in other studies, however quantitative profile was significantly different, which emphasizes the important influence of the extraction method and applied solvents [9,18,19]. In addition, the parameter regarding the total content of catechins and gallic acid in all extracts was very different (10.2–748 mg/200 mL), which shows how important the method of extraction is, as well as the application of an appropriate solvent. The highest was observed when using ASE and ethanol:water mixture and the lowest in the case of using ultrasound extraction and ethyl acetate.

Based on the obtained results, it can be concluded that all the extraction techniques studied showed similar efficiency in the extraction of the tested catechins. However, larger differences occur when using different solvents. The results herein obtained are in agreement with the previous findings reported by Perva-Uzunalic et al. analyzing the effect of different temperatures and solvents on the efficiency of extraction of catechins from green tea who saw that EGCG was the dominant compound extracted from green tea in different solvent and temperature systems. They also showed that pure methanol and ethanol better extract catechins from the tested raw material, while in the case of acetone the addition of 20% of water significantly improved the extraction efficiency. They also observed that the use of organic solvents and their aqueous mixtures increases the efficiency of extraction of catechins from green tea compared to pure water [15]. Results of the present study are in agreement with these findings. The presented results show that of the solvent systems used, ethyl acetate turned out to be the solvent that extracted the least amount of polyphenol compounds, while the extract most rich in polyphenols was obtained using ethanol/water mixture. The present study shed also a new light on the extraction efficiency of particular catechins, when using different extraction models. From the data presented in the Table 2 it appears that the most effective solvent system for the extraction of EGCG and EGC was by far the mixture of ethanol and water. Application of maceration or ASE gave similar results, which were not statistically significant. However, in the case of ultrasound extraction, the mixture of acetone and water was proved to be the best for the extraction of EGCG. This solvent system was also proved to be the most efficient for ECG for all extraction techniques. Regarding the concentration of C, EC and GA the most effective was hot water, which was proved by PCA analysis. In the case of GA, combination of ultrasound maceration or ASE with water resulted in statistically significant higher extraction efficiency in comparison to simple maceration. Lan-Sook and co-workers applied Response Surface Methodology (RSM) for the optimization of extraction efficiency of phenolics from green tea. The predicted optimal conditions for the highest antioxidant activity and minimum caffeine level were found at 19.7% ethanol, 26.4 min extraction time and 24.0 °C extraction temperature [20]. They also revealed that the ratio of (EGCG + ECG)/EGC was identified a major factor contributing to the antioxidant activity of green tea extracts, which is in agreement with results of the present study.

The results obtained in a Folin–Ciocalteu test, which was used to determine the total content of polyphenolic compounds in the samples tested and may also characterize the antioxidant activity of the extracts, were confirmed by the chromatographic determinations described above. Ethyl acetate is by far the least effective extractant, since only small amounts of catechins were detected in the samples tested, regardless of the extraction technique used. Unlike ethyl acetate, among all the solvents tested, a mixture of ethanol/water was the most effective in delivering extracts richest in catechins. Application of simple maceration and ASE gave comparable effects using individual solvents. A slightly lower degree of extraction was noted using ultrasounds, except for extraction using mixture of acetone and water, in which this method proved to be the most effective, as these extracts contained the largest amount of polyphenolic compounds regarding F–C determinations. Results of antioxidant activity of extracts obtained using various extraction models including F–C method were presented in Table 3. Obtained results of F–C analysis were in agreement with our previous data for green tea infusions [9], showing that this product is very rich in polyphenols and therefore presents high antiradical activity, similarly to *Vaccinium meridionale*—a berry known for its high polyphenols content and antioxidant activity (up to 724.24 mg gallic acid/L) [21]. A very high concentration of phenolics, determined using F–C reagent, was also revealed in the cryoconcentrates obtained from fruits of another berry-maqui-berries (*Aristotelia chilensis*), which contained up to 4311.8 mg gallic acid/100 g [22]. Bilberry and blackberry pomace extracts were also proved to contain high amounts of protocatechuic acid (3.36–35.18 mg g^−1^) and gallic acid (9.57–31.98 mg g^−1^) and therefore presented high antioxidant activity, confirmed with electron spin resonance (ESR) spectroscopy [23]. In addition to tea or different species of berries, coffee also may be characterized by high content of phenolics and significant antioxidant properties. Recently, Beder-Belkhiri and co-workers revealed that filtered Algerian coffee contained up to 690 mg of gallic acid equivalents/100 g and Turkish coffee exhibited the highest antiradical activity, with 73.34% towards DPPH radical [24].

However, not only is the high content of compounds with antioxidant properties such as polyphenols a crucial factor to take into consideration for polyphenols to exert their health beneficial properties, but equally important is also the actual bioavailability of these compounds (which may be influenced by other nutrients, temperature, pH or gastric digestion) [25,26].

The antioxidant capacity of the obtained green tea extracts was additionally evaluated using DPPH and expressed both in percentage of the radicals scavenged by the extracts and Trolox equivalents. Overall, the extracts produced by ultrasounds were characterized by lower antioxidant activity in comparison to other techniques. It can be assumed that ultrasounds negatively affect the stability of polyphenolic compounds, therefore the antioxidant potential of these extracts is lower, which was also shown in previous studies [27,28]. In addition, Sun and co-investigators—who were studying the influence of sonification on various parameters of fresh apple juice—noticed a significant decrease of phenolic content and antioxidant activity measured by oxygen radical absorption capacity assay (ORAC). They concluded that, indeed, application of ultrasound increased the extraction of polyphenols, but later free radicals which were produced by sonification increased the degradation of polyphenols [29]. On the other hand, several studies revealed that application of ultrasounds significantly increased concentration of polyphenols and antioxidant activity of the extracts [30,31,32,33,34]. However, according to Setyaningsih et al. 2016, an increase in antioxidant potential and phenolics concentration is observed to a temperature of 60–70 °C; above this level, a significant deterioration of polyphenols may be observed, which also decreases antioxidant activity of the samples [27]. Zapata and co-investigators—who studied the effect of pH, temperature and time of extraction on the antioxidant properties of *Vaccinium meridionale*—observed that an increase of temperature resulted in more efficient extraction of polyphenols. However, anthocyanins were degraded above 80 °C and within 20 min of processing [21]. In addition, in this method, ethyl acetate was characterized by the weakest extraction capacity, and the extracts obtained using this solvent present activity below 10% (Trolox equiv. <0.5 mM/L), and these samples were rated as inactive. Therefore, their activity was not marked in Figure S1. Only in the case of ethyl acetate extracts obtained using ASE, some minor antiradical activity was observed, and these values were presented in Figure S1. In general, the results of antioxidant activity of the obtained extracts were in agreement with chromatographic determinations. The highest ability to deactivate the DPPH radical was demonstrated by extracts obtained using a mixture of ethanol and water (1:1 *v/v*), as well as a mixture of acetone and water (5:1 *v/v*). The high antioxidant capacity of these extracts can be associated with the high content of EGCG and EGC as revealed by chromatographic results.

### 2.3. PCA Analysis

**Figure 2:**
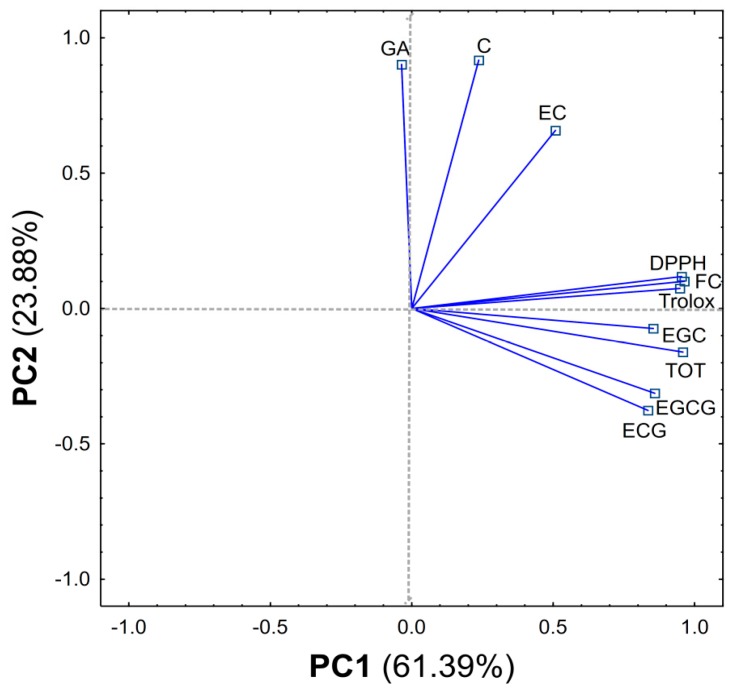
Loadings of first two components of PCA (Principal Component Analysis), explaining together 85.27% of information in the obtained dataset.

**Figure 3:**
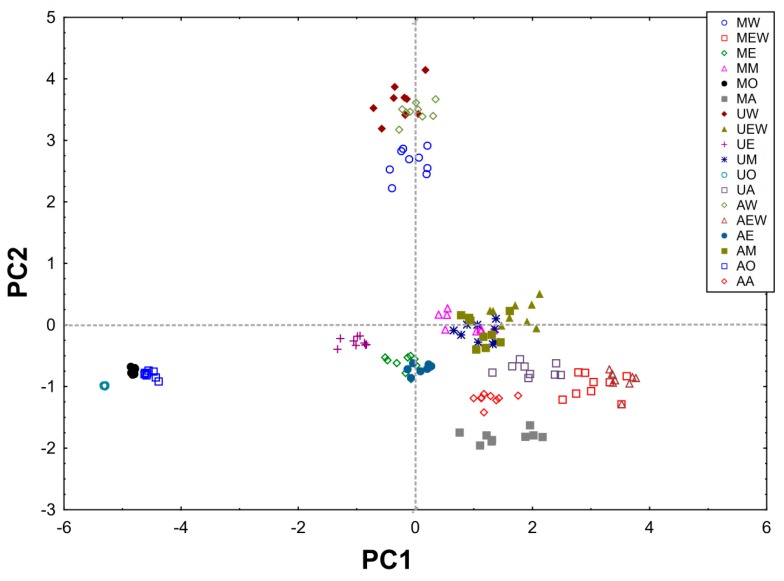
Scores of first two principal components of PCA, explaining together 85.27% of information in the obtained dataset.

From the obtained results, a matrix made of columns (content of compounds, F–C method, DPPH, Trolox equivalent) and rows (type of extract) was created and subjected to PCA analysis. The PCA carried out explains 85.27% of the variability in the first two principal components (61.39% and 23.88%, respectively) (Figure 2 and Figure 3).

The first component (PC1) is related to the overall extraction efficiency and shows the differences between MO, UO, AO and other methods MW, MEW, ME, MM, MA, UW, UEW, UE, UM, UA, AW, AEW, AE, AM, AA. In addition, it can be concluded that the best methods were AEW and MEW. The PC1 component shows one group containing compounds found in large quantities in green tea (EGC, EGCG, ECG and TOT-sum of all compounds) and the activity of the tested extracts (DPPH, F–C method, Trolox equivalent) which are strongly and positively correlated (Table S1).

The second component (PC2) reveals another group (GA, C, EC) which contains compounds found in a small amount in green tea compared to the other ingredients tested. In addition, the PC2 component separates the MW, UW, AW methods (all using water as solvent) as the best methods for C, EC and GA extraction from among the solvents used (Figure S1).

## 3. Materials and Methods

### 3.1. Plant Material

Green tea used in the study was an original product cultivated in Sri Lanka, which was purchased from a professional tea shop in Poland. This specific tea was chosen for extraction studies based on our previous research that revealed high quality of this product regarding catechin composition and antioxidant activity [9].

### 3.2. Chemicals

The solvents used for LC–MS analyses (acetonitrile, formic acid and water) were of spectroscopic grade and were purchased from J. T. Baker (Center Valley, PA, USA). The solvents used to perform extraction (ethanol, water, ethyl acetate, acetone, methanol) were of analytical grade and were obtained from Avator Performance Materials (POCH, Gliwice, Poland). Folin–Ciocalteu reagent, sodium carbonate, DMSO and citric acid were bought from Stanlab (Lublin, Poland). Standards of catechins (EGC, EGCG, ECG, C and EC), gallic acid, DPPH (2,2-diphenyl-1-picrylhydrazyl) and Trolox were purchased from Sigma-Aldrich (St. Louis, MO, USA).

### 3.3. Extraction

#### 3.3.1. Simple Maceration and Ultrasound Assisted Maceration

Green tea leaves were first ground in a ceramic mortar and then 2.0 g was weight into conical glass flasks and macerated for 10 min in a water bath with 200 mL of fresh solvent specific for each extraction performed at a temperature of 80 °C or at its boiling point (the values of boiling point were measured for each extractant and are presented in Table 4). The specific weight/solvent ratio was typical for conventional tea brewing methods and based on previous studies [9,35]. The extraction time of 10 min was influenced by the former studies of the authors. In this work the extraction time was, however, prolonged from 3 to 10 min to recover higher quantity of catechins extracted by traditional extraction techniques, like simple maceration [35,36]. Ultrasound assisted maceration was performed for 10 min in ultrasonic bath (EMAG Emmi-55HC-Q) operated with the ultrasonic power of 300 W and frequency of 45 kHz at the specified temperature settings (Table 4).

#### 3.3.2. Accelerated Solvent Extraction (ASE)

The portions of 2.0 g of green tea leaves were placed in a stainless steel cells and extracted for 10 min using ASE 100 Accelerated Solvent Extractor (Dionex, Sunnyvale, CA, USA) using the same conditions and temperature settings as in the case of maceration. The purge time was set at 30 s, the flush volume at 50% and the pressure was maintained at ca. 100 bar.

Subsequently all the extracts were filtered through study filter, their pH was decreased to 3.2 using citric acid (only for LC–MS determinations), as described previously [9], and their volume was made up to 200 mL with a specific solvent. Extracts which were subjected to chromatographic determinations were filtered using 0.22-µm nylon syringe filter (Cronus, Gloucester, UK), diluted 5-times with LC–MS grade water and/or acetonitrile and subjected to LC–MS analysis.

### 3.4. LC-ESI-Q-TOF-MS Analysis of Green Tea Extracts

Chromatographic determinations were performed using Agilent LC system (Series 1200, Agilent Technologies, Santa Clara, CA, USA) composed of the degasser (G1322A), a binary pump (G1312C), a PDA detector (G1315D) and an autosampler (G1329B), and combined with an ESI-Q-TOF-MS 6500 Series mass spectrometer (Agilent Technologies, Santa Clara, CA, USA). The separation of the extracts was performed in a 30-min long gradient elution method (Table 5) using an Agilent Technologies Zorbax RP 18 150 mm × 2.1 mm; 3.5-μm chromatographic column.

The time of analysis was set at 30 min, the post-run at 5 min and the flow rate at 0.2 mL/min. The spectra were recorded in both: negative and positive ionization modes, within the *m/z* range of 50–1000. The following mass spectrometer settings were used: capillary voltage of 4000 V, skimmer voltage 65 V, fragmentation voltage 120 V, the gas and sheath gas (nitrogen) temperatures of 350 and 400 °C each, and their flow rates of 12 L/min, respectively. The nebulization pressure was 35.0 psi g. And the injection volume was set at 20 µL of each reference compound and tea infusion. Identification of each compound was performed based on mass spectra and retention times of standards, literature data and on-line mass libraries (Metlin), whereas the quantitative analyses were obtained using the 7-point calibration curves plotted for each investigated compound. The data were analyzed by the Agilent MassHunter Qualitative Analysis Navigator program B.08.00.

The method was previously validated and optimized for determination of catechins and gallic acid in different types of tea, which was described elsewhere, and slightly modified for the purpose of this study [9,14].

### 3.5. Determination of Total Phenolic Content (TPC)

The content of total phenols, which is very often described as one of the antioxidant parameters [37], was determined using a modified protocol by Singleton and co-workers [38]. Briefly, 0.5 mL of each extract was mixed with 30 mL of distilled water and 2.5 mL of Folin–Ciocalteu (F–C) reagent. Subsequently 7.5 mL of 20% sodium carbonate was added, the mixture was filled up with distilled water to a final volume of 50 mL and was incubated in dark for 2 h. To plot the calibration curve, water solutions of gallic acid in the range of 50–500 mg/L were added instead of samples and were subjected to the same procedure. The absorbance was read at 760 nm in 1-cm cuvettes using UV-Vis Thermo Fisher Scientific Evolution 300 Spectrophotometer (Thermo Electron Scientific Instruments LLC, Madison, WI, USA). Obtained results were expressed as mg of GA per 1 L of each extract.

### 3.6. Free Radical Scavenging Activity (DPPH Test)

A previously described protocol [39] was used after a few modifications. The stock solution of DPPH was prepared by dilution of 10 mg of DPPH free radical in 100 mL of methanol. Obtained extracts were diluted with appropriate solvent (1 + 3 *v/v*) and were mixed with 3.9 mL of DPPH stock solution. After the reaction reached the plateau value (AC_t_) (max. after 30 min) the absorbance was read at a wavelength of 515 nm in 1-cm cuvettes using UV-Vis spectrophotometer against the blank (methanol). The following equation: I (inhibition) [%] = [(AC_0_/AC_t_)/AC_0_] × 100, where AC_0_ represents initial absorbance of DPPH, was used to calculate free radical scavenging activity of the extracts, expressed as the amount of DPPH radical scavenged by each extract. Additionally, antiradical potential was expressed as Trolox equivalent. Therefore, Trolox standard water solutions were prepared in a concentration of 0.5–15 mM/L and were used using the same protocol as the test extracts.

### 3.7. Statistical Analysis

All determinations were performed in triplicates. Statistical analysis was performed using Microsoft Excel 2010, Statistica 13.5 (StatSoft, Tulsa, OK, USA) and Graph Pad Prism 7 (Graph Pad Software, San Diego, CA, USA). Data were analyzed for normal distribution using the Shapiro–Wilk test. The statistical significance between results obtained for different types of extraction were analyzed using two-way ANOVA (with two qualitative factors) followed by Tukey’s test. Pearson’s correlation analysis was used to check the relationship between the studied variables. To reduce the number of variables and to detect the structure of relationships between variables, principal component analysis (PCA) was used. All results are presented as mean ± standard deviation (SD). The level of statistical significance was set at *p* ≤ 0.05.

## 4. Conclusions

The obtained results revealed which extraction solvents were responsible for the highest antiradical capacity of the obtained tea extracts and for the concentration of individual catechins and gallic acid present in green tea. The highest value of total catechins was detected in the extracts obtained by the ASE method and simple maceration, using a mixture of ethanol and water in a ratio of 1:1 (*v/v*) and the lowest—in the extracts obtained by means of ultrasound assisted extraction in which ethyl acetate was used as a solvent. EGCG was the catechin found at the highest concentration in most of the extracts, except UEW, MO and AO in which EGC or ECG were the major ones. EC and GA were found in the lowest concentrations in all extracts. The total content of polyphenols in the extracts obtained by various methods was similar, with slightly smaller quantities found in the samples obtained by ultrasound based extraction. Regarding the solvent system selection, the most efficient was the mixture of ethanol and water (1:1 *v/v*) and the lowest efficiency was obtained for ethyl acetate. In general, the extraction efficiency was clearly associated with the solvent system used and not with the extraction technique applied. Antiradical activity of the extracts was strongly correlated with the content of catechins and the samples obtained by ASE and maceration were characterized by the highest sum of polyphenols in the DPPH and Folin–Ciocalteu assays.

## Figures and Tables

**Table 1:** Applied extraction conditions in the study.

Extraction Type	Solvent	Code
Simple maceration	Water	MW
	Ethanol:water (1:1 *v/v*)	MEW
	Ethanol	ME
	Methanol	MM
	Ethyl acetate	MO
	Acetone:water (5:1 *v/v*)	MA
Ultrasound assisted maceration	Water	UW
	Ethanol:water (1:1 *v/v*)	UEW
	Ethanol	UE
	Methanol	UM
	Ethyl acetate	UO
	Acetone:water (5:1 *v/v*)	UA
Accelerated solvent extraction (ASE)	Water	AW
	Ethanol:water (1:1 *v/v*)	AEW
	Ethanol	AE
	Methanol	AM
	Ethyl acetate	AO
	Acetone:water (5:1 *v/v*)	AA

**Table 2:** Concentration of catechins and gallic acid and sum of all investigated compounds in the obtained extracts. Each value represents mean ± SD. Means not sharing the same letter are significantly different at *p* ≤ 0.05.

Sample Code	C	EC	ECG	EGC	EGCG	GA	TOT
Maceration (mg/200 mL)							
MW	26.20 ^h^	9.99 ^ef^	61.03 ^d^	79.52 ^cd^	119.11 ^c^	31.09 ^i^	326.94 ^c^
SD	1.63	1.50	4.27	5.88	13.18	3.60	18.45
MEW	5.24 ^b^	5.38 ^c^	132.89 ^h^	175.78 ^g^	404.33 ^i^	15.49 ^h^	739.11 ^i^
SD	0.40	0.82	14.53	9.19	45.40	2.23	40.00
ME	6.64 ^bc^	5.93 ^cd^	88.81 ^fg^	86.96 ^d^	160.00 ^ed^	0.39 ^a^	348.73 ^c^
SD	0.88	0.69	9.35	5.78	14.40	0.05	20.72
MM	9.19 ^fg^	9.85 ^ef^	91.22 ^fg^	142.78 ^ef^	177.22 ^ef^	6.20 ^cde^	436.46 ^d^
SD	1.25	0.73	8.05	18.84	15.94	0.77	33.01
MO	1.06 ^a^	0.17 ^a^	29.73 ^b^	2.43 ^a^	24.76 ^ab^	10.16 ^fg^	68.31 ^b^
SD	0.11	0.01	2.13	0.18	3.36	1.20	4.27
MA	7.47 ^cde^	−	164.00 ^i^	134.00 ^e^	274.78 ^gh^	0.41 ^a^	580.66 ^h^
SD	0.82	−	10.10	17.85	14.82	0.05	27.09
Ultrasound extraction (mg/200 mL)							
UW	28.18 ^i^	12.25 ^g^	54.34 ^cd^	80.70 ^d^	117.33 ^c^	47.72 ^j^	340.53 ^c^
SD	2.76	1.57	7.34	8.01	8.97	6.35	10.89
UEW	5.91 ^bc^	14.32 ^h^	101.67 ^g^	200.56 ^h^	155.00 ^de^	7.35 ^def^	483.81 ^ef^
SD	0.36	1.53	7.38	12.39	12.37	0.75	20.78
UE	7.11 ^c^	5.88^cd^	77.68 ^ef^	77.86 ^cd^	139.89 ^cd^	7.62 ^abc^	316.04 ^c^
SD	0.65	0.77	5.38	5.02	11.11	0.54	9.85
UM	9.10 ^def^	10.60 ^f^	98.10 ^g^	158.89 ^fg^	181.78 ^ef^	2.48 ^ab^	460.95 ^de^
SD	0.86	1.21	8.95	6.72	6.69	0.17	14.90
UO	0.13 ^a^	0.03 ^a^	3.17 ^a^	0.52 ^a^	5.44 ^a^	0.92^ab^	10.2 ^a1^
SD	0.02	0.001	0.36	0.05	0.34	0.13	0.67
UA	7.33 ^cd^	8.93 ^e^	122.11 ^h^	141.67 ^e^	262.89 ^g^	−	542.92 ^g^
SD	0.62	0.82	12.49	12.13	11.67	−	18.20
ASE (mg/200 mL)							
AW	23.60 ^g^	14.46 ^h^	63.87 ^de^	63.00 ^c^	118.78 ^c^	47.30 ^j^	331.00 ^c^
SD	2.34	1.13	8.25	6.65	8.78	4.52	9.13
AEW	6.80 ^bc^	7.29 ^d^	125.89 ^h^	193.00 ^h^	406.00 ^i^	9.02 ^efg^	748.01 ^i^
SD	0.96	0.98	14.60	19.25	33.63	1.59	27.67
AE	5.84 ^bc^	2.48 ^b^	91.22 ^fg^	72.81 ^cd^	139.33 ^cd^	3.33 ^abc^	315.02 ^c^
SD	0.57	0.23	6.30	6.89	18.42	0.27	20.49
AM	9.73 ^f^	13.87 ^h^	123.78 ^h^	135.44 ^e^	199.67 ^f^	0.47 ^a^	482.96 ^ef^
SD	0.72	1.56	14.60	14.06	25.11	0.06	31.10
AO	1.10 ^a^	0.19 ^a^	45.11 ^c^	1.72 ^a^	41.77 ^b^	11.97 ^gh^	101.84 ^b^
SD	0.15	0.02	6.59	0.17	5.07	1.19	8.02
AA	6.08 ^bc^	4.46 ^c^	151.67 ^i^	34.73 ^b^	304.22 ^h^	4.41 ^bcd^	505.47 ^f^
SD	0.55	0.38	10.68	4.02	22.74	0.26	21.57

LOD and LOQ for C (0.42 and 1.26 ng/mL), EC (0.42 and 1.26 ng/mL), ECG (0.42 and 1.26 ng/mL), EGC (0.42 and 1.26 ng/mL), EGCG (0.46 and 1.38 ng/mL) and GA (0.52 and 1.56 ng/mL), respectively [9,14].

**Table 3:** Antioxidant activity of the obtained extracts. Each value represents mean ± SD. Means not sharing the same letter are significantly different at *p* ≤ 0.05.

Sample Code	F–C Method (mg/L)	DPPH (%)	Trolox Equivalent (mM/L)
Maceration			
MW	631.78 ± 16.13 ^de^	66.63 ± 4.99 ^cde^	2.31 ± 0.23 ^cdef^
MEW	828.89 ± 40.95 ^kl^	78.83 ± 6.47 ^fg^	2.86 ± 0.28^hi^
ME	613.00 ± 17.35 ^d^	62.24 ± 4.08 ^c^	2.16 ± 0.20 ^bcd^
MM	748.33 ±18.77 ^hij^	64.48 ± 3.96 ^cd^	2.24 ± 0.21 ^cde^
MO	127.11 ± 11.01 ^b^	<10	<0.5
MA	670.22 ± 32.69 ^efg^	67.70 ± 9.44 ^cde^	2.39 ± 0.44 ^cdefg^
Ultrasound extraction			
UW	614.22 ± 21.36^d^	59.89±7.47^bc^	2.09±0.27 ^bc^
UEW	780.00 ± 51.09^jk^	72.08±6.29^def^	2.59±0.28 ^efgh^
UE	485.33 ± 19.76^c^	51.32±5.08^b^	1.79±0.14 ^b^
UM	757.33 ± 19.51^ij^	64.91±4.34^cd^	2.27±0.21 ^cde^
UO	72.00 ± 5.54^a^	<10	<0.5
UA	794.00 ± 27.78^jkl^	75.63 ± 7.14 ^ef^	2.74 ± 0.35 ^gh^
ASE			
AW	660.44 ± 28.22 ^def^	65.91 ± 6.00^cd^	2.27 ± 0.24 ^cde^
AEW	842.22 ± 18.97 ^l^	85.50 ± 3.28 ^g^	3.19 ± 0.12 ^i^
AE	722.33 ± 81.01 ^gh^	75.08 ± 6.03^ef^	2.70 ± 0.29 ^fgh^
AM	696.56 ± 28.49 ^fgh^	60.54 ± 4.81^c^	2.08 ± 0.19 ^bc^
AO	71.34 ± 5.09 ^a^	14.58 ± 1.87^a^	0.61 ± 0.05 ^a^
AA	760.78 ± 35.13 ^ij^	71.60 ± 4.42^def^	2.54 ± 0.21 ^defgh^

**Table 4:** Boiling point of the solvent systems used in the study.

Solvent System	Boiling Point (°C)
Ethanol:water (1:1 *v/v*)	80
Ethanol	72
Methanol	68
Ethyl acetate	72
Acetone:water (5:1 *v/v*)	60

**Table 5:** Mobile phase composition.

Time (min)	Solvent A (0.1% Formic Acid) (%)	Solvent B (2% Acetic Acid in Acetonitrile) (%)
0	90	10
10	60	40
12	60	40
17	5	95
20	90	10
30	90	10

## Supplementary Materials

The following are available online, Figure S1: Graphs showing the content of phytochemical compounds (C, EC, ECG, EGC, EGCG, GA, TOT), F–C equivalent, DPPH and Trolox equivalent. Each value represents mean ±SD. Means not sharing the same letter are significantly different at *p* ≤ 0.05. Table S1: Correlation between the content of phytochemical compounds and F–C method, DPPH and Trolox equivalent. Table S2: The MS/MS spectra of all compounds quantified in the obtained extract in the negative ionization mode, together with a sample total mass chromatogram.

## Abbreviations

GA	gallic acid
EGCG	epigallocatechin gallate
EGC	epigallocatechin
ECG	epicatechin gallate
EC	epicatechin
C	catechin
MW	maceration with water
MEW	maceration with ethanol:water (1:1 *v/v*)
ME	maceration with ethanol
MM	maceration with methanol
MO	maceration with ethyl acetate
MA	maceration with acetone:water (5:1 *v/v*)
UW	ultrasound extraction with water
UEW	ultrasound extraction with ethanol:water (1:1 *v/v*)
UE	ultrasound extraction with ethanol
UM	ultrasound extraction with methanol
UO	ultrasound extraction with ethyl acetate
UA	ultrasound extraction with acetone:water (5:1 *v/v*)
AW	Accelerated Solvent Extraction (ASE) with water
AEW	ASE with ethanol:water (1:1 *v/v*)
AE	ASE with ethanol
AM	ASE with methanol
AO	ASE with ethyl acetate
AA	ASE with acetone:water (5:1 *v/v*)
