# Supplementary material for: The Role of Extracting Solvents in the Recovery of Polyphenols from Green Tea and Its Antiradical Activity Supported by Principal Component Analysis
Source: Molecules. 2020 May 6;25(9):2173. doi: 10.3390/molecules25092173 (PMC7248709; doi:10.3390/molecules25092173)
Supplement: Supplementary file 1 [file molecules-25-02173-s001.pdf]

SUPPLEMENTARY FILE

The role of extracting solvents in the recovery of polyphenols from green tea and its antiradical activity supported by Principal Component Analysis

Wojciech Koch [1]*, Wirginia Kukula-Koch [2], Marcin Czop [3], Paweł Helon [4] and Ewelina Gumbarewicz [5]

[1] Chair and Department of Food and Nutrition, Medical University of Lublin, 4a Chodźki Str., 20-093 Lublin, Poland (W.K.)
[2] Chair and Department of Pharmacognosy, Medical University of Lublin, 1 Chodźki Str., 20-093 Lublin, Poland; virginia.kukula@gmail.com (W.K.-K.)
[3] Department of Clinical Genetics, Medical University of Lublin, Radziwiłłowska 11 Str., 20-080 Lublin, Poland; marcin.czop@umlub.pl (M.C.)
[4] Branch in Sandomierz, Jan Kochanowski University in Kielce, Schinzla 13a Str., 27-600 Sandomierz, Poland; phelon@ujk.edu.pl (P.H.)
5 Department of Biochemistry and Molecular Biology, Medical University of Lublin, 1 Chodźki Str., 20-093 Lublin, Poland; ewelina.gumbarewicz@umlub.pl (E.G.)
* Correspondence: kochw@interia.pl; Tel.: +48-81-448-7143

Table S1. Correlation between the content of phytochemical compounds and F-C method, DPPH and Trolox equivalent.

	C	EC	ECG	EGC	EGCG	GA	TOT
**F-C**	0.317***	0.576***	0.757***	0.794***	0.756***	0.009	0.873***
**DPPH**	0.093	0.243**	0.543***	0.603***	0.634***	-0.075	0.733***
**Trolox**	0.034	0.187*	0.547***	0.605***	0.655***	-0.097	0.741***

* $p \leq 0.05$, ** $p \leq 0.01$, *** $p \leq 0.001$

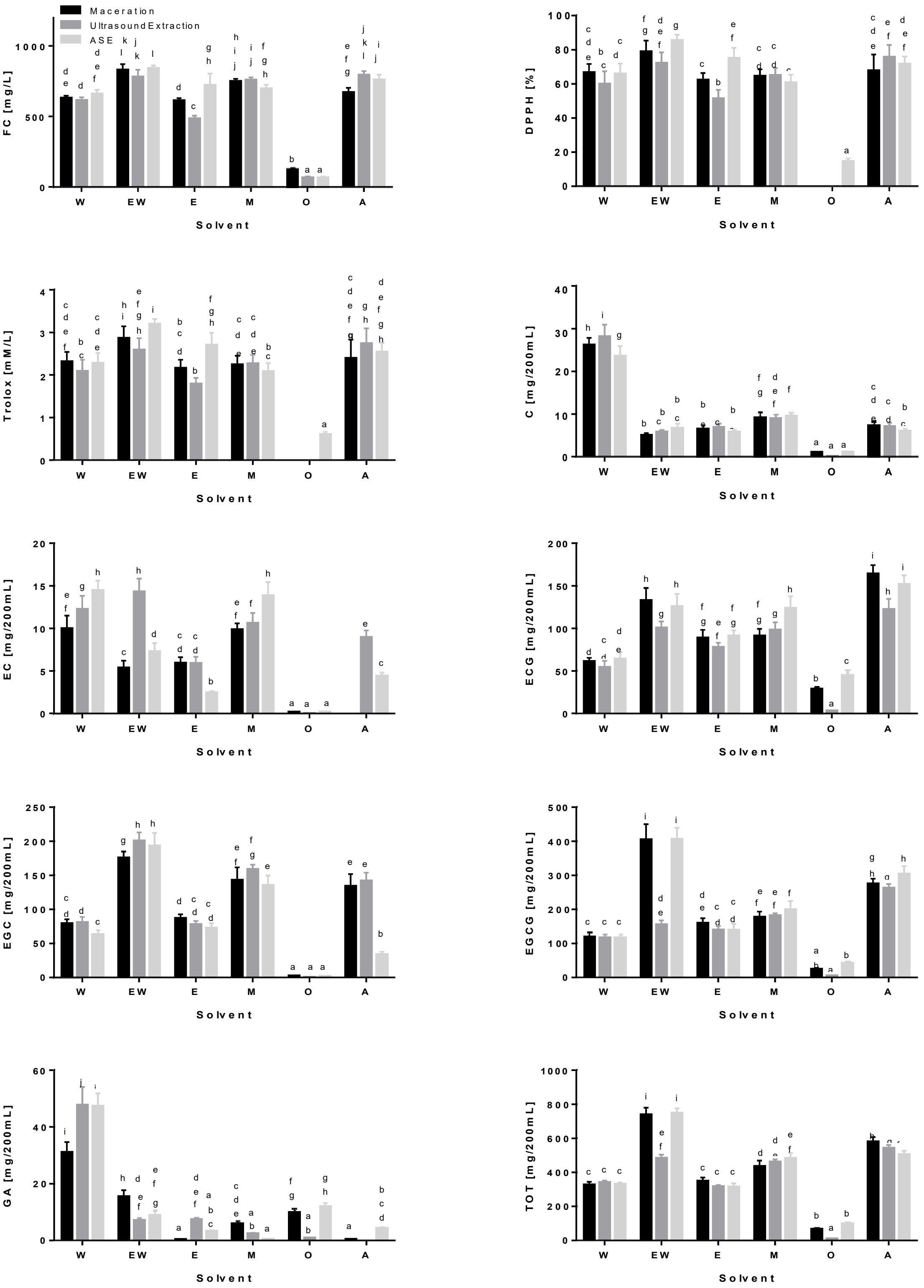

Figure S1. Graphs showing the content of phytochemical compounds (C, EC, ECG, EGC, EGCG, GA, TOT), F-C equivalent, DPPH and Trolox equivalent. Each value represents mean ±SD. Means not sharing the same letter are significantly different at $p \leq 0.05$.

Table S2. The MS/MS spectra of all compounds quantified in the obtained extract in the negative ionization mode, together with a sample total mass chromatogram.

<table>
<tr><td colspan="2">The total ion chromatogram (TIC) of the tea extract</td></tr>
<tr><td colspan="2">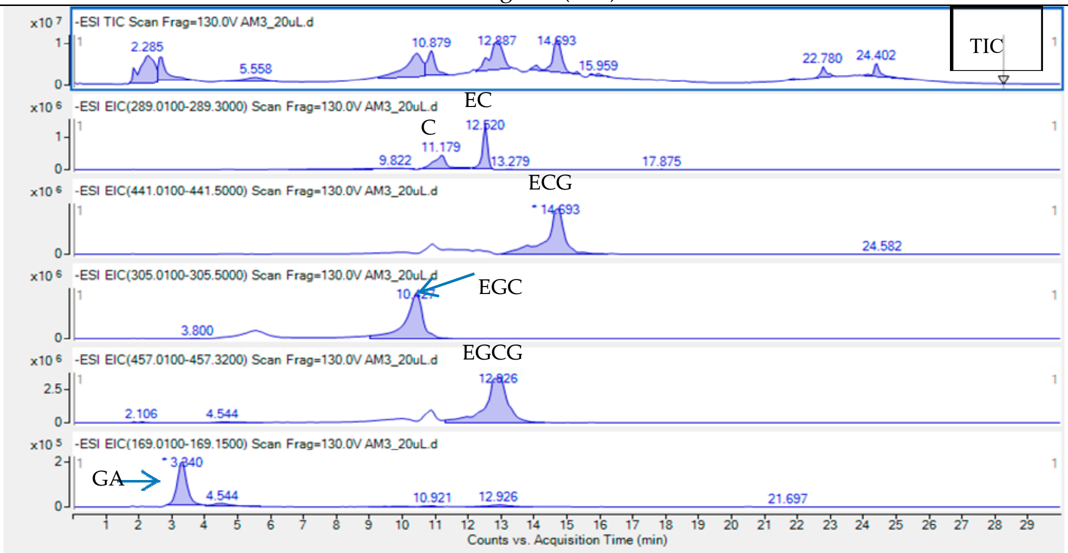
</td></tr>
<tr><td>Gallic acid (MW=169)</td><td>Epicatechin (MW=289)</td></tr>
<tr><td>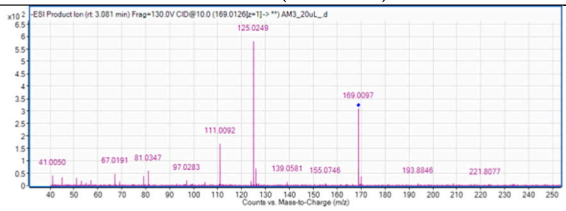</td><td>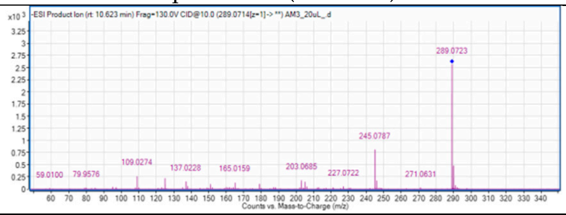</td></tr>
<tr><td>Catechin (MW=289)</td><td>Epicatechin gallate (MW=441)</td></tr>
<tr><td>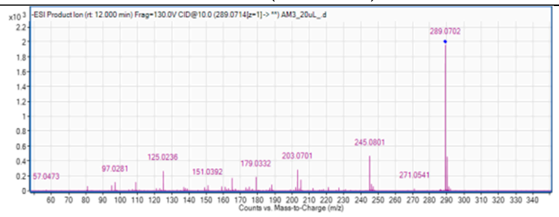</td><td>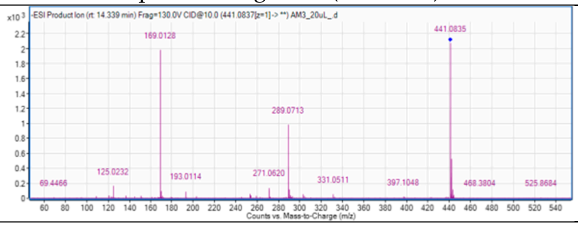</td></tr>
<tr><td>Epigallocatechin gallate (MW=457)</td><td>Epigallocatechin (MW=305)</td></tr>
<tr><td>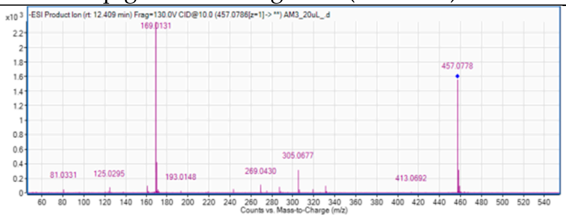</td><td>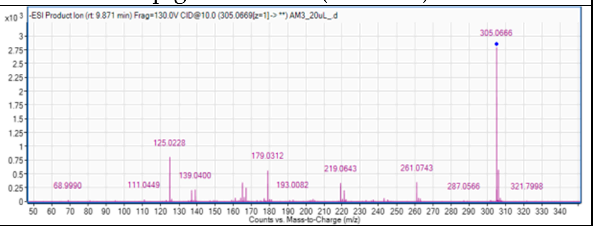</td></tr>
</table>
